# Instructors’ Views on and Experiences with *Last Aid Courses* as a Means for Public Palliative Care Education—A Longitudinal Mixed-Methods Study

**DOI:** 10.3390/ijerph22071117

**Published:** 2025-07-15

**Authors:** Georg Bollig, Sindy Müller-Koch, Erika Zelko

**Affiliations:** 1 Department of Anesthesiology, Intensive Care, Palliative Medicine and Pain Therapy, Helios Klinikum, 24837 Schleswig, Germany; 2 Department of Palliative Medicine, Faculty of Medicine and University Hospital, University of Cologne, 50924 Cologne, Germany; 3 Last Aid Research Group International (LARGI), 24837 Schleswig, Germany; erika.zelko@jku.at; 4 Letzte Hilfe Deutschland gGmbH, 24837 Schleswig, Germany; sindy.mueller-koch@lerchenberg-wittenberg.de; 5 Nursing home am Lerchenberg, 06886 Wittenberg, Germany; 6 Institute for Palliative Medicine, Medical Faculty of University Maribor, 2000 Maribor, Slovenia; 7 Institute for General Medicine, Medical Faculty, Johannes Keppler University, 4040 Linz, Austria

**Keywords:** palliative care, public awareness, end-of-life care, Last Aid Course, public palliative care education, instructor, facilitator, death literacy, grief literacy, mixed methods, social space

## Abstract

Background and aims: The Last Aid Course (LAC) has been established to enhance the discussion about dying, death and grief and to raise the public’s awareness of palliative care. The aim of this study was to explore the views and experiences of German Last Aid Course instructors with the LAC as means for Public Palliative Care Education (PPCE), including their opinion about the course content and format and practical aspects of teaching in different settings. Methods: A longitudinal mixed-methods approach was used to explore the views and experiences of the Last Aid Course instructors over a period of five years. Social space orientation was used as the framework for the data analysis. Results: The LAC participants felt empowered after the LACs. Continuing development was a characteristic of the LAC project. The positive effects of the LACs included empowerment and positive interactions between the instructors and participants. In addition, the LACs had a positive impact on all five principles of social space orientation. Conclusions: LACs can contribute to raising public awareness about dying, death, grief and palliative care and empower people to participate in caring for those who are serious ill, dying and grieving.

## 1. Introduction

The Last Aid Course (LAC) and the so-called public knowledge approach to palliative care were first described by Bollig in 2008 [1]. The international Last Aid Course concept includes a standardised curriculum to educate the public about dying, death, grief and palliative care to increase both the death literacy and grief literacy of the public. These LACs have four modules, 45 min each, and are usually taught by two certified Last Aid Course instructors in a classroom setting with 6 to 20 participants. The methods used in the LACs include lectures, practical demonstrations and practical training as well as reflection on and discussions about death and dying in general. The LACs were started in 2015 in Germany, Norway and Denmark and, to date, have been introduced in 23 countries. Table S1 provides an overview of the Last Aid Course modules and their main themes and contents. According to the public knowledge approach, Last Aid should be as well known as first aid and everybody should learn to provide Last Aid [1]. Everybody should be able to provide first aid in emergency situations like traffic accidents, heart attacks, etc. In some countries, such as Germany, people are obligated to provide first aid by law [2]. Emergency care is thus usually provided through cooperation of the people in the community and the professionals working in the Emergency Medical and Health Care Services. This cooperation allows people to receive help from members of the public if needed. A similar cooperation between health care professionals and the public should be a normal part of public health palliative care (PHPC) and end-of-life care including research [1,3,4,5,6]. In addition to first aid everyone should therefore be able to provide basic Last Aid measures including simple palliative care measures to relief suffering and contacting professional palliative care from the health care system because end-of-life care is as much everyone’s business as first aid is [1,5,6]. To prepare people to perform these measures, they need to be given both education and motivation to participate in caring for others including end-of-life care [1,3]. Since most people prefer to die at home and palliative care resources are limited, there is a need to involve people in the community in palliative care provision [5,6,7,8]. A German study from Westphalia showed that home was registered as the place of death in 21.3% of cases in 2017, while the majority of deaths (51.8%) took place in hospitals [9]. This study revealed a decrease in home deaths from 27.8% in 2001 to 21.3% in 2017 [9]. Internationally, the number of deaths occurring at home ranged from 17.8% in Sweden to 30.7% in the USA [9]. In Germany, the percentage of patients receiving specialised palliative care from mobile palliative care teams was 13% on average, and ranged from 6 to 23% depending on the part of the country [10]. These findings indicate a need for public involvement in palliative care and end-of-life care in the community as the resources and staffing of professional health services are not sufficient to serve all the people who want to die at home. Last Aid International aims to educate the public and to encourage public discourse about serious illness, dying, death, grief and palliative care by implementing Last Aid Courses for children, teenagers and adults in different countries [11]. Since 2015, Public Palliative Care Education (PPCE) through Last Aid Courses (LACs) has been introduced in a number of countries to educate people about palliative care and to enhance the public discourse on death and dying [8,11]. Similar to first aid courses, the Last Aid Course aims to educate people to enable them to participate in the provision of basic healthcare in the community; it also provides a meeting place for people to talk openly about death, dying and grief and to reflect on their own attitudes towards these topics [1,8,11,12,13,14,15,16,17]. It took more than ten years from the first description of a Last Aid Course as part of the so-called public knowledge approach for the implementation of palliative care in the community in 2008, to the introduction of the course to 23 countries including European and South American countries, Canada, Singapore and Australia [1,8,9,10,11]. An International Last Aid working group and Last Aid Research Group International (LARGI) have been established and a number of publications and lectures have been published about Last Aid Courses in different countries [1,3,4,8,11,12,13,14,15,16,17,18,19]. This work has shown that Last Aid Courses for the public are feasible and well accepted by both adults and children aged 7–17 years [11,12,13,14,15,16,17]. Between 2017 and 2024, five national German Last Aid Conferences and four international Last Aid conferences (one in Sønderborg, Denmark, in 2019; one online conference in 2020 during the COVID-19 pandemic; one in Maribor, Slovenia, in 2022; and the 4th International Last Aid Conference in Cologne, Germany, in 2024) have been held [11,16,18]. During the COVID-19 pandemic, Online Last Aid Courses (OLACs) for the public were successfully introduced starting in April 2020 [14]. At present, more than 6000 Last Aid Course instructors have been trained and more than 120,000 people have participated in Last Aid Courses in Germany, Austria and Switzerland [11]. The German non-profit, non-governmental Last Aid organisation Letzte Hilfe Deutschland gGmbH celebrated the 10th anniversary of the introduction of LACs in Germany in May 2025. Last Aid Courses have become widely recognised since the first LACs held in Norway, Germany and Denmark in 2014/2015. It has been suggested that Last Aid Courses can encourage people to participate in end-of-life care in the community and can be seen as an educational basis for developing compassionate communities; they could also encourage community action in the field of public health palliative care and end-of-life care [1,8,13,16,17,19].

**Aim of the study** To explore the views and experiences of Last Aid Course instructors from Germany with the course as means for Public Palliative Care Education including their opinion about course content, format, practical aspects and the effects of teaching in different settings.

To date, most studies on LACs delved into the views and experiences of the Last Aid Course participants. Thus, to obtain more insights and a richer picture of the experience of participating in LACs, the views and experiences of the Last Aid Course instructors are needed. Furthermore, the results of this study could contribute to the future adaptation and further development of the Last Aid Course content and curriculum and the implementation of the worldwide Last Aid Course project in different countries. We used the five-step framework of social space orientation to explore the needs and effects of LACs as a means for PPCE.

## 2. Materials and Methods

### 2.1. Framework of This Study

Social space orientation is a concept that places people’s living space at the centre of action. It is about recognising, using and improving living conditions, resources and social networks. Fürst and Hinte describe social space orientation as not only involving the physical conditions of a space, but also the social interactions and cultural dynamics that occur in that space [20]. According to Fürst and Hinte, social space orientation is a response to the challenges of individualisation and social change [20]. It requires a differentiated analysis of people’s life situations and aims to strengthen the self-help potential of individuals and communities. In addition, Grunwald and Tiersch emphasise that social space orientation also focuses on the participation of those affected [21]. When people are involved in the design of their social space, sustainable solutions to social problems can be developed. This means that life-world orientation is an approach that focuses on people’s everyday experiences and social realities to provide support. Löw’s approach of ‘social spaces’ offers a valuable perspective for understanding the dynamics of spaces and social practices [22]. She argues that spaces are not only physical features, but also socially constructed places that are characterised by social relationships and interactions. This view supports the idea of social space orientation by emphasising the interactions between individuals and their social environment. On the other hand, Bourdieu focuses on the social and cultural capital that individuals possess in the various social spaces [23]. Bourdieu’s concept of ‘habitus’ is particularly relevant in relation to social space orientation, as it describes how individual actions are influenced by social structures. The consideration of habitus and capital enables a deeper understanding of how social inequalities are reproduced in specific spaces. Social space orientation is an important approach that emphasises the importance of social space for individual and community life. Based on the ideas of Grunwald and Tiersch [21], Löw [22] and Bourdieu [23], Fürst and Hinte [20] offer five valuable perspectives for analysing the dynamics in social spaces and developing practical approaches for improving people’s living conditions:

1. Resource orientation: The principle of resource orientation emphasises that within a social space, the challenges, strengths and resources of the individuals and communities concerned should be considered.
2. Participation: Participation is a central element of social space orientation. It is postulated that people in the social space should be actively involved in shaping their living conditions.
3. Networking: The networking of the actors within a social space is of great importance for the success of social space-orientated approaches. Network actors, be they social institutions, neighbours or initiatives, can work together to develop solutions and use resources more efficiently.
4. Sustainability: Sustainability in social space-orientated work implies that measures do not merely offer short-term solutions, but aim for long-term changes. This requires continuous reflection and adaptation of strategies and services in order to promote the residents’ ability to help themselves.
5. Contextuality: The principle of contextuality states that social space-orientated measures must be adapted to the specific circumstances of a space.

Linking the five principles of social space orientation with the concepts of Löw [22] and Bourdieu [23] enables a deeper understanding of the social dynamics in a space. These approaches not only promote the analysis and design of living spaces, but also active co-design by the people living there. By taking these five principles into account, a social space-orientated practice can be developed that meets the needs of the communities and improves the living conditions in the long term.

In this study, social space orientation was chosen as the framework for the analysis of the qualitative data from the questionnaires and focus group discussions because of the possible implications that the LACs might have on the social space and social interactions in the community. Using this framework and the five principles enabled the researchers to examine the effects of the LACs on the different aspects of the social space.

### 2.2. Study Design, Data Collection and Analysis

This study was based on a longitudinal mixed-methods design [24,25,26] in order to provide a rich description of the views and experiences of Last Aid Course instructors regarding the courses and their contents over a period of five years. Last Aid Course instructors were invited to answer questionnaires and to participate in focus group discussions between 2018 and 2023. Questionnaires including both quantitative and qualitative data were used to collect data from certified German speaking Last Aid Course instructors. The questionnaires were based on previous studies on Last Aid Course participants using questionnaires and interviews [14,19] and on informal reports of experiences from Last Aid Course instructors and participants. The key themes and questions that were included in the questionnaires can be found in Supplementary Table S2. The questionnaires (which were written in German) are available upon request to the corresponding author. In addition to the questionnaires, focus group discussions were used to explore the experiences of the Last Aid Course instructors in greater depth [25,26]. The focus group discussions were held in person or as a video meeting via Zoom. The focus group discussions were recorded digitally. The interviews were transcribed verbatim by Amberscript [27], with revisions made by S.M.-K. and G.B. after checking the audio files and transcripts. Box 1 shows the questions for the focus group discussions from the interview guide.

Box 1 **Questions from the interview guide for the focus group discussions.**

- What are your experiences with Last Aid Courses?
- In which setting and location have the Last Aid Courses been held?
- What kind of practical exercises did you use in the Last Aid Course?
- May the Last Aid Courses serve to recruit people to become hospice volunteers?
- How can Last Aid Courses contribute to compassionate communities?

The qualitative study design used in the current study was interpretive description as described by Thorne and colleagues [28,29,30]. The research team (S.M.-K., G.B. and E.Z.) started the analysis process after reviewing and revising the transcripts made by Amberscript. The researchers independently coded the qualitative data. After the preliminary coding, the codes and themes were discussed and an agreement on the final codes and themes was reached. Repeated reading of the qualitative data and repeated discussions between the authors were used to question the results and to validate the findings [25]. Table S3 (in the Supplementary Materials) shows the details of the analysis process.

Interpretive description was used for the analysis and presentation of the qualitative data from the questionnaires and focus group interviews [28,29,30]. Interpretive descriptions provide a valuable framework for investigating phenomena that cannot be easily measured quantitatively. It also promotes a comprehensive understanding of people’s subjective experiences. With this in mind, the categories were formed through a multi-stage discussion process between the researchers. As part of a triangular process, the different categories were related to each other and validated. In this context, the researchers reflected on their own role as researchers. The fact that subjective interpretations and assumptions influence the data was taken into account [31]. The reporting of the results followed the current recommendations and guidelines for mixed-methods studies from the Enhancing the Quality and Transparency Of health Research (EQUATOR) network and Consolidated Criteria for Reporting Qualitative Research (COREQ) for reporting qualitative research [24,32,33]. The quantitative data collected in this study included the total number of LACs taught by the participants (who were all certified Last Aid Course instructors), the number of LAC instructors, age and gender of the informants. Quantitative description was used to analyse and describe the quantitative data from the questionnaires.

### 2.3. Setting, Participants and Sample Selection

Questionnaires were used to collect both quantitative and qualitative data from German-speaking Last Aid Course instructors who attended at least one German Last Aid Conference or a national Last Aid Trainer meeting: the 2. German Last Aid Symposium, 5 October 2018 in Kassel; the 3. German Last Aid Symposium in Munich, 29 October 2019 or the Last Aid Trainer meeting in Schleswig 26–27 July 2022. All LAC instructors attending at least one of the above meetings were invited to participate in the study. As the meetings were all held in Germany all participants did speak German.

In addition to the data collected from the questionnaires focus group interviews were held during the 2. and 3. German Last Aid symposia and the Last Aid Trainer meeting in Schleswig in 2023. All the participants in the current study were certified Last Aid Course instructors or certified Last Aid Course trainers. Last Aid Courses must be run by a team of two certified instructors and one of them has to a nurse or a doctor working in palliative care to ensure that the team has specialised palliative care competence and the expertise to answer medical questions from the audience. In order to become a certified Last Aid Course instructor, one must have practical experience in the field of palliative care. This includes people such as nurses, physicians, social workers, priests and hospice volunteers. LAC instructors with a large amount of experience teaching LACs to the public and teaching in general can become Last Aid trainers. The certified LAC trainers educate LAC instructors. All the researchers are LAC trainers themselves and have extensive experience teaching LACs. Some of the participants knew the researchers from the LACs, or training or meetings connected to the LACs.

The inclusion criteria for participation in the present study were as follows:

- Certified German-speaking LAC instructor or LAC trainer;
- Participation in at least one German Last Aid symposium or Last Aid Trainer meeting.

All people who did not meet the above inclusion criteria were excluded from participating in this study. All the participants received information about this study and its purpose and were informed that participation in this evaluation was voluntary and that they could choose not to complete the questionnaire or participate in the focus groups if they did not want to. All the participants provided oral informed consent. The data for the longitudinal study were collected over a five-year period between October 2018 and September 2023. Due to the worldwide COVID-19 pandemic, no data were collected between 2020 and 2022 because focus group discussions could not be held.

## 3. Results

The results are presented in the following subsections: the quantitative and qualitative data from the questionnaires are presented in Section 3.1 and Section 3.2, respectively, and the results from the focus group interviews are presented in Section 3.3. In total, 758 LACs were run by 102 instructors; 57 of the included instructors had taught fewer than 5 LACs and 14 participants had no experience in teaching LACs.

### 3.1. Quantitative Data from the Questionnaires

Questionnaires were distributed during three large meetings of Last Aid Course instructors within the study period. Table 1 provides an overview of the participants from these three meetings.

### 3.2. Qualitative Data from the Questionnaires

A summary of the qualitative data and quotations from the questionnaires are shown in Table 2, organized by the four main themes and thirteen subthemes that were identified through the analysis of the qualitative data. The main themes from the qualitative data derived from the questionnaires were: informal peer advice; impact; challenges and symptom awareness.

As part of the questionnaire the instructors were asked about their wishes to the Last Aid project team. The most important wishes include: a regular revision of the LAC presentation; to keep the high quality of the materials; information material for the participants; handouts for the participants; support for the instructors and new developments (see Table S4 in the Supplementary Materials).

### 3.3. Qualitative Results from the Focus Group Interviews

**Figure 1:**
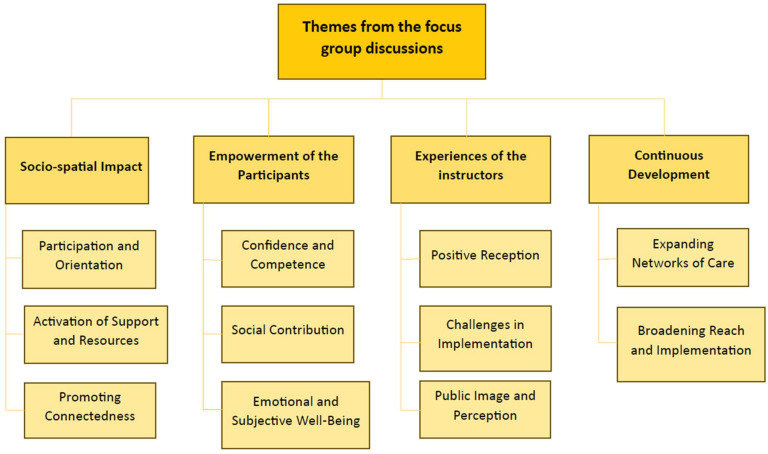
Themes from the focus group discussions.

Four central themes and eleven sub-themes were identified in the analysis of the available data from the transcripts of the focus group interviews (see Figure 1).

The different themes shown in Figure 1 are explained in more detail below and illustrated with quotes from the focus group participants.

(1) **Socio-Spatial Impact**

*Participation and Orientation*

The LACs had an impact on two levels that complemented each other and can be seen as essential building blocks for a caring community: one level focuses on individual, case-specific needs, while the other aims to promote general, non-case-specific engagement:

‘And how do we strengthen our cohesion there again. It’s a great help, not just to go into the specialist area and say, ‘Here and there you get someone, but to encourage the citizens to be there for each other’.’

(Trainer interview, p. 14)

The multidimensional impact of the LACs in the social sphere was due to various effects. At the individual level, the principles of need, interest and will orientation was demonstrated:

‘People who are currently in the care situation or want to look retrospectively, did I do it right back then with my mum. Yes, it’s just great that we reach so many people with it and that people give consistently positive feedback.’

(Trainer interview, p. 1)

*Activation of Support and Resources*

The life-world orientation manifests itself in a variety of aspects, including an increasing need for support and relationships in living spaces:

‘…the aim is to encourage people to simply continue to live their lives, their families or whatever relationship systems they may have, as before.’

(Group discussion Kassel 2, p. 13)

Organising LACs promotes life-enhancing resources through intensive networking:

‘I think you can say that the networks expand through last aid courses because you build up contacts that you wouldn’t otherwise build up in this way, and last aid courses are also a good vehicle for getting into dialogue with each other.’

(Trainer interview, p. 8)

In this regard, it should be noted that the activation of support potential is an essential component. The course participants were encouraged to contribute to the community with their individual strengths and talents:

‘… by empowering the people who attend our courses and encouraging them, bring yourselves with your talent, with your strengths, so look in the neighbourhood, in the neighbourhood.’

(Trainer interview, p. 15)

*Promoting Connectedness*

The opportunity for exchange and dialogue was found to open up spaces for social participation and promote connectedness:

‘We also introduced our regional structures to people who needed more information and initiated contacts if there was a need for them.’

(Group discussion Kassel 1, p. 10)

The results suggest that the courses contributed to the expansion of caring communities through their socio-spatial effect by promoting the experience of being connected in a social environment.

‘I can think of the church communities and organisations that say it’s important that we do a course like this so that we can get to grips with the topic, deal with it personally, because it might affect someone somewhere in our society at some point. And how do we strengthen our cohesion again?’

(Trainer interview p. 14)

Another aspect was the promotion of networking in a social environment through which resources can be tapped into both on a case-specific and non-case-specific basis.

‘I run a course at the AWO /Arbeiterwohlfahrt) service centre for the staff there, who in turn use the neighbourhood helpers. And that’s where a network proves its worth…’

(Trainer interview, p. 6)

(2) **Empowerment of the Participants**

Experiencing self-efficacy and empowerment was a central element in the LACs, which taught various methods that could be implemented.

*Confidence and Competence*

The participants’ ability to act was strengthened by teaching them how to utilise their own resources in the care of seriously ill and dying people:

‘I also experience that fear of contact with the dying is reduced, i.e., the idea that you are not allowed to sit in bed with them, and you are not allowed to hold them from behind if they have difficulty breathing, and you are also allowed to lie in bed with the dying person.’

(Group discussion Kassel 2, p. 11)

The participants had the opportunity to share their experiences with the other participants and the trainers. By enhancing their capabilities, reducing their fears and imparting knowledge through practical exercises, the LACs induced a significant increase in the participants’ sense of safety:

‘So the best feedback for me was actually from two women who said afterwards that this four-hour course had given them such a sense of security in accompanying a dying person that they felt well prepared, always ready for the next symptom that comes and had a total calmness in the accompaniment, or how you can achieve that with four hours, it’s great.’

(Group discussion Kassel 2, p. 4)

*Social Contribution*

The LACs made a major social contribution by focusing on the needs, interests and living environments of the participants:

‘And how do we strengthen our cohesion there too. It’s a great help, not just that it goes into the technical side of things, but that you bring someone in and encourage people to be there for each other.’

(Trainer interview, p. 14)

*Emotional and Subjective Well-Being*

The reduction in fears and the expansion of options for action were essential elements, particularly in the context of the practical exercises. The discussions between the participants and the exchange with the course leaders served to strengthen the subjective experience and emotional stabilisation of the course participants:

‘… and then there are also personal experiences that come up in the course, and I think that’s very important, not just the course content, but that we meet people in person, that there is space and time for them to report and that we incorporate this and learn from each other together.’

(Trainer interview, p. 1)

(3) **Experiences of the Instructors**

*Positive Reception*

The LACs were initially held in adult education centres and hospice and palliative care locations. With new venues and diversified course formats, new target groups are being included and the demand continues to rise. During the continuing development process of Last Aid in recent years, special courses for kids, teens, and health care professionals were established as well as the Last Aid Course in Easy Language for people with learning and cognitive disabilities and Last Aid Course Diversity, which provides more time to discuss the impact of diverse backgrounds on dealing with death and dying. The increased demand for LACs is described as positive:

‘At our Hospice Academy, last-aid courses are more or less like hot cakes, they are always fully booked. Both in our programme and as in-house training courses, we only have good experiences with good feedback.’

(Trainer interview, p. 1)

The reasons for the increased demand vary and can be explained by different motives, for example, a need for information:

‘… they sort of soak it up like a sponge and are really, really interested.’

(Trainer interview p.4)

The increasing awareness of Last Aid Courses is also described as positive experience:

‘So for me that shows that it’s getting a very high level of penetration more and more in the sense of awareness, of ‘this is worthwhile’. And it’s no longer just our job to advertise, but to keep the ball rolling.’

(Trainer interview, p. 8)

*Challenges in Implementation*

The increased demand often resulted in administrative challenges with regard to the number of participants:

‘We are completely overrun and always have a waiting list.’

(Group discussion Kassel 2, p. 1)

another challenge is:

‘Well, I’ve also had two mishaps in terms of registration, once at the parish in Mörlebach, where the deacon advertised it and then went on holiday for three weeks. And people registered at the parish office, and then I came to hold the course and there were 36 people there.’

(Trainer interview, p. 8)

In addition to coping with organisational tasks, spatial conditions were also an obstacle:

‘… and then you only find out about the pitfalls on site, which means that you still have to improvise some things.’

(Group discussion Kassel 2, p. 4)

Financing was also perceived as a challenge, especially with regard to the time spent working:

‘Sure we get donations, but if you think about it, that’s not enough to finance our working hours.’

(Group discussion Kassel 1, p. 5)

*Public Image and Perception*

The public perception of LACs was described as a significant problem. On the one hand, the increased demand indicated a positive view of LACs, but on the other hand, there were pronounced reservations about them because people did not know what a LAC is about:

‘They are interested in doing it, so the demand seems to be there among the trainees. (…) But there are reservations about clearly stating what it is.’

(Trainer interview, p. 10)

‘(…)Last Aid is still seen as competition, not at all as a basic information event at a low-threshold level or, but always as competition to a longer Palliative Care Course and to other specialised training courses. (…) I think that is sometimes the case, perhaps a small image problem that we have as last aid.’

(Trainer interview, p. 16)

(4) **Continuous Development**

*Expanding Networks of Care*

The LACs led to the recruitment and empowerment of new volunteers:

‘It also happens with us that one or two people who have made this career through an information evening perhaps and then attend a last aid course and then become volunteers.’

(Trainer interview, p. 2)

*Broadening Reach and Implementation*

Knowledge of regional services and care structures was found to be of fundamental importance for the support of seriously ill people and their relatives from volunteers:

‘Even people who needed more information, where we then also introduce our regional structures, initiate contacts…’

(Group discussion Kassel 1, p. 10)

The established contacts also led to new support from volunteers:

‘Or requests for accompaniment, we have that again and again. When the relatives are at home during the course, we come into contact with them and that sometimes leads to counselling.’

(Group discussion Kassel 1, p. 10)

In order to initiate this kind of extended contact, the Last Aid Courses are held in different areas of the community. New venues will enable the course to reach new target groups of participants and lead to a more heterogeneous composition of participants that differs significantly from those usually found in such courses.

‘For example, we were approached by a yoga centre. These are completely different people who go to the course.’

(Trainer interview, p. 7)

The positive experiences with running the LACs in places in the centre of communities, such as the DASA working world exhibition, can be cited as evidence:

‘So we reach a lot of people there, and the best thing about the courses is that they are really colourful (…) people who are currently in a care situation or want to look back (…).’

(Trainer interview, p. 1)

The increased demand for LACs led to a diversification of the course offerings, which, based on the training needs identified, led to the development of new course formats, for example, a format for professional caregivers:

‘We developed the last aid course for professional careers, which runs over eight hours. Then, we tested it in a facility, so we trained the whole building, the day-to-day carers with the Müller course and the nursing staff with our newly developed course (…).’

(Group discussion Kassel 2, p. 7)

This development of the learning system shows that with the continuous differentiation of the LACs and their increasing application to new target groups, new network structures of hospice and palliative care are being established, which increases the courses’ reach and impact in the social space.

## 4. Discussion

The results of the current study support earlier findings that LACs are well accepted by the public and that many participants feel empowered to participate in end-of-life care in the community after LAC participation [12,14,15,17,19]. Considering that in 2016, globally, over 20 million people were in need of end-of-life palliative care [34], palliative care is believed to be a public health issue and public health care interventions could enable communities to support dying people [3,34,35,36,37,38]. Talking about dying, death and grief is still a taboo in many parts of society and the public is lacking in death literacy as well as awareness and knowledge of and the skills for palliative care [39,40,41,42,43,44]. Last Aid Courses for the public can be a part of the implementation of compassionate communities and aim to increase both death and grief literacy and to empower people to participate in palliative care [1,8,19].

Social space orientation was used as the framework for the analysis of the data in the current study. A number of positive effects of LACs in the social space were found. The LACs were shown to have a positive influence on all five aspects of social space orientation, which is about recognising, using and improving living conditions, resources and social networks:

1. Resource orientation: During the LACs, the participants learn new facts but are also reminded to use their own knowledge and skills and to trust their intuition. According to Fürst and Hinte [20], it is ‘[…] about empowerment, a sense of self-efficacy, about empowerment or self-authorisation.’

2. Participation: During the LACs, people become aware that they can become part of a compassionate community and actively participate in palliative care in the community in cooperation with healthcare professionals, palliative care teams and other organisations and people, thereby shaping their living conditions.

In Beckers’ words, ‘Against this background, non-family and neighbourhood relationships and social contacts are important factors influencing the subjective quality of life, especially for those older people who have no available family support potential in their social environment’ [45].

3. Networking: In the LACs, the participants learn about existing networks in their local community. By reflecting on the available options they can identify network partners that they already know, and also learn about previously unknown options for support and networking, encouraging them to support each other and become part of a caring community. This enables the participants to cooperatively develop solutions with others within the community. This improved networking also contributes to a more efficient use of resources and, according to Bleck, Reißen, and Knoopp, can thus help to “… enable people to age well and independently, even if they require care” [46].

4. Sustainability: Through participation in an LAC, people are able to reflect on their strategies to cope with dying, death and grief. They gain practical experience in talking about these themes without any negative consequences, making many of them feel empowered and willing to care for others in the future. This contributes to the adaptation of strategies and services in the compassionate community that promote the people’s ability to help themselves. According to Kellehear, this demonstrates that end-of-life care is everyone’s business [5]. Rüßler and Stiel add that such thinking “… takes into account the diverse skills of older people, their independence and self-determination, and involves them as responsible actors in a participatory manner” [47].

5. Contextuality: The local networks, options for support and measures to care for dying and grieving people can be adapted to the needs of the local community after participation in an LAC. Many participants feel encouraged to start advance care planning and to prepare for the care of seriously ill and dying people in their local community while taking into account the local and regional options for palliative care. In this context, Becker emphasises that the ‘… significant increase in the number of older people and those in need of care, and the simultaneous fragility of family care, which is characterised by the increasing fragility of intra-family networks due to growing mobility, point to the ever-increasing importance of a living environment in which citizens do not live unconnectedly side by side, but rather stand up for each other and take responsibility [45]. This corresponds to the idea of caring communities described by Kellehear [5,6].

Our results support similar results from earlier research on LAC especially on the following themes: 

*Empowerment*—LACs lead to empowerment of the participants as they are encouraged to talk openly about the topics of dying, death and grief and cooperate with health care professionals in palliative care provision in the community. These results are similar to those of previous research on LACs [3,12,14,17,19]. Furthermore, the LACs contributed to the recruitment of volunteers for hospice services, similar to the findings of the study by Teixeira and colleagues [48]. The importance of volunteers in palliative care through being there for the dying person has been already emphasised [49]. Through education and motivation of citizens, LACs can also contribute to the implementation and networking of compassionate communities. LACs may contribute to raise awareness for end-of-life care and palliative care in the public space [16]. Previous studies have suggested that LACs encourage people to engage in end-of-life care and can lead to a feeling of empowerment [12,16,17]. The need to raise awareness about palliative care has also been recognised by Biondo and colleagues who developed a “Understanding Palliative Care” online educational module for defining and promoting palliative care to a public audience [43]. According to the findings from Giehl et al., LACs not only provide a safe space to discuss death and dying, but they also empower and encourage the participants to engage in end-of-life care provision [42].

*Interaction of instructors and participants*—Many instructors described teaching LACs as very rewarding and touching because of the open discussions and the participants’ personal stories. The LAC instructors often experienced a positive reception and positive feedback from the course participants, and many of them described a feeling of personal fulfilment. Time management, including the time to respond to the participants’ needs and coordinate with the paired instructor, was described as a challenge by the instructors. These experiences are similar to the experiences of LAC instructors of an online LAC during the COVID-19 pandemic [14].

*Continuing development of Last Aid*—The continuing development of Last Aid is often described as important by the instructors. Last Aid, as a learning system, has been continuously adapted to the needs of the participants, the instructors and the people in the communities in recent years. Within the study period, new LAC course formats had been created based on the needs and wishes of the course participants and the LAC instructors, as e.g., Last Aid in Easy Language, Last Aid Professional and Last Aid Diversity [50]. Scientific research and expert working groups have established these new course formats in response to the special needs of different audiences and the results of research on the Last Aid Project.

The LAC also helps to expand local networks of care and to broaden the reach of Public Palliative Care Education by offering courses in new locations like schools, yoga centres, museums, etc. These findings have not been described in previous studies but have been discussed at international Last Aid conferences since 2019. LACs are a place for laypersons from the local community to meet professionals from palliative care services (e.g., hospices, palliative care units in hospitals or mobile palliative care teams) to discuss death, dying and grief; they also offer a platform for learning about local and regional options for palliative care, thus contributing to expanding networks. Some LAC instructors report increased trust in them as professionals and their palliative care services after LACs because the participants get to know them personally during the course.

### Practice Implications and Future Research Directions

The Lancet Commission of Death and Dying has suggested that we should bring death back into life [51]. This can be achieved through LACs as they encourage discussions about death, dying and grief, raising awareness of palliative care and empowering people to participate in palliative care provision in the community. LACs also provide knowledge about local and regional palliative care services and options for support. Furthermore, LACs contribute to expanding and improving networks of support. In recent years, different Last Aid Course formats have been developed based on research. Thus, LACs should be introduced and implemented in more regions and countries around the world. Further research should aim to investigate (1) the long-term effects of LACs on all participants and specifically the participants who are caring for others at home; (2) the possible contribution of LACs to increasing the percentage of deaths occurring at home; and (3) the effects of different course formats.

### Strengths and Limitations

This study is the first longitudinal, mixed-methods investigation into the experiences of Last Aid Course (LAC) instructors over a five-year period. The longitudinal approach can be seen as a major strength of this study as the data collection occurred at several time points in different years. This allowed for the detection of changes over time in the data. All the authors are certified LAC instructors and have conducted a number of LACs as instructors. This can be seen as a strength since the researchers have first-hand experience and insights on conducting and implementing LACs. In order to identify any influence that this experience had on their role as a researcher, the authors used group discussions to reflect on their roles. While the chosen methodology provided valuable insights, several limitations must be acknowledged. First, the primary reliance on qualitative data may restrict the generalizability of the findings. Additionally, there is a potential for bias in both the data collection and data analysis. Another limitation is the absence of randomisation of the participants, which may have introduced selection bias. We cannot completely exclude the unconscious bias in our research, as some of the participating instructors knew that the researchers were fellow instructors. Furthermore, the COVID-19 pandemic occurred during the study period, which may have influenced both participation and the experiences reported. Future studies could strengthen the validity and reliability of the results by triangulating qualitative findings with additional quantitative data, particularly from focus group interviews.

## 5. Conclusions

The results of our study underline the role of the Last Aid Course as an approach for PPCE and for strengthening compassionate communities. The results suggest that the LAC is a useful tool to increase both death literacy, grief literacy and knowledge about palliative care of the public.

## Figures and Tables

**Table 1:** Demographic and quantitative data from the questionnaires.

Meeting	2. German Last Aid Symposium in Kassel in 2018	3. German Last Aid Symposium in Munich in 2019	Last Aid Trainer Meeting in Schleswig in 2022
Number of participants	77	118	9
Number of returned questionnaires	37	72	9
Return rate	48%	61%	100%
Age (range and mean)	n.o.	40–78 (57)	42–67 (50)
Female	n.o.	60	5
Male	n.o.	11	4
Other	n.o.	0	0
No information about gender in questionnaire	n.o.	1	0
Number of Last Aid Courses Taught (range and mean)	0–20 (5.8)	0–20 (5.3)	3–60 (27)

n.o. = no information.

**Table 2:** Qualitative data from the questionnaires.

Theme	Subtheme	Quotations from the Questionnaires
**Informal Peer Advice**	Organisational Aspects and Environment	“‘In the midst of life’—football stadium, communities, museum; everywhere hospice/ambulatory hospice service.” “If it is prepared with ‘love,’ the location doesn’t matter.”
	Engagement and Interaction	“Very moving personal disclosures with grief reactions. Every time, at the end, realizing that the participants feel touched.”
	Variety in Teaching Methods	“Reporting case studies and personal experiences, having an emergency kit for the fridge, and engaging in practical skills such as hand massage, touch, and oral care are all important components. The presentation of films and books, alongside the use of varied methods, helps prevent fatigue and keeps participants engaged.”
**Impact**	Instructors’ Experiences	“The gratitude of a participant who, after the course, felt confident enough to let her husband die at home.” “Human support! For example, a lady had a bad feeling because her mother no longer allowed herself to be touched in her final hours. Once she learned that this was normal and why, she felt relieved.”
	Community and Recruitment	“Recruiting volunteer helpers/hospice volunteers. Participants feel encouraged and empowered to accompany the dying in the future, contributing to helping people lose their fear of dying and engage with the topic.” “Regional connections and available offerings.”
	Participants Empowerment	“The sudden realisation of a participant: If I do everything the same way as with my baby, then it’s actually right—and I already know how to do it!” “A participant’s statement after the course: Now I am much less afraid.” “A mother and daughter, who cared for and accompanied their husband/father with glioblastoma, left the course feeling very empowered without dominating the course.”
	Time Management	“A limited timeframe restricts the variety of methods.” “Point out the time limit from the beginning. Offer alternatives and advice for later.”
	Technical	“Rooms with good technical equipment and no long, narrow spaces.”
**Challenges**	Participant-Related	“Different participants with varying levels of knowledge. Participants who dive into their personal struggles and take up a lot of space.” “Uncertain group dynamics.”
	Facilitation and Coordination	“Collaboration with a co-leader who struggles to be concise.” “Catering to the needs of all participants.”
	Applying Practical Skills	“Oral care with different materials and flavours. Everyone is allowed to try oral care (possibly with a neighbour).” “Kinesthetic positioning, and gentle stroking from the ear downwards to stimulate saliva production.”
	Alternative Teaching Methods	“Drawing cards on the topic of grief and reading them aloud.” “Breathing through a straw (as a simulation of breathlessness).”
**Symptom awareness**	Diversity of symptoms	“Address the specificities of dying in people with dementia.” “Assign symptoms to the dimensions of Total Pain.”

## Data Availability

The data presented in this study are available upon request to the corresponding author. The data are not publicly available due to privacy restrictions.

## Supplementary Materials

The following supporting information can be downloaded at: https://www.mdpi.com/article/10.3390/ijerph22071117/s1, Table S1: Contents of the Last Aid Course—Care for Seriously Ill and Dying People at the End of Life; Table S2: Key themes and questions included in the questionnaires; Table S3: Detailed steps of the analysis process for the qualitative data; Table S4: Instructors wishes to the project team.

## Abbreviations

The following abbreviations are used in this manuscript:

LAC	Last Aid Course
LARGI	Last Aid Research Group International
OLAC	Online Last Aid Course
PHPC	Public Health Palliative Care
PPCE	Public Palliative Care Education
