# Supplementary material for: Instructors’ Views on and Experiences with Last Aid Courses as a Means for Public Palliative Care Education—A Longitudinal Mixed-Methods Study
Source: Int J Environ Res Public Health. 2025 Jul 15;22(7):1117. doi: 10.3390/ijerph22071117 (PMC12294638; doi:10.3390/ijerph22071117)
Supplement: Supplementary file 1 [file ijerph-22-01117-s001.zip › ijerph-3678610-supplementary.pdf]

<u>Table S1:</u> Contents of the Last Aid Course - Care for seriously ill and dying people at the end of life

Module Nr.	Topic	Course Content
Module 1	Dying as a normal part of life	• Welcome and introductions<br>• First Aid and Last Aid<br>• What you can do to care<br>• The process of dying
Module 2	Planning ahead	• Networks of Support<br>• Making decisions<br>• Medical and ethical aspects<br>• Advance care planning<br>• Advance Directive<br>• Medical Power of Attorney
Module 3	Relieving suffering	• Typical problems and symptoms<br>• Caring/relieving suffering<br>• Nutrition at the end of life<br>• How to comfort
Module 4	Final goodbyes	• Saying goodbye/final farewell rituals<br>• Funeral and various forms of burials<br>• Grieving is normal<br>• Grief and ways of grieving<br>• Questions, Comments

Table S2 Key themes and questions included in the questionnaires:

- Number of Last Aid Courses taught by the instructor
- Recommendation for the presentation of the course (insider tips)
- Where is your emphasis in the different modules?
- In which setting/location does the course work best?
- The most important experiences in a Last Aid Course
- Which practical measures do you teach beside mouth care as part of the course?
- Your wishes to the Last Aid project team

Table S3: Detailed steps of the analysis process of the qualitative data

1. S.M.-K., G.B. and E.Z. read the transcripts and familiarised themselves with the data
2. S.M.-K. and G.B. independently identified preliminary codes and themes
3. S.M.-K., G.B. and E.Z. compared and discussed the preliminary codes and themes
4. S.M.-K. coded all the material according to the preliminary codes and themes
5. S.M.-K., G.B. and E.Z. revised the preliminary codes and themes
6. S.M.-K., G.B. and E.Z. discussed the revised codes and themes and agreed on the final codes and themes
7. S.M.-K., G.B. and E.Z. checked the transcripts in order to question the findings
8. S.M.-K., G.B. and E.Z. discussed the findings and themes and agreed about the interpretation of the data

Table S4: Instructors wishes to the project team

**Wishes to the project team**	*2. German Last Aid Symposium in Kassel*	*3. German Last Aid Symposium Munich*	*Last Aid Trainer meeting Schleswig*
A revised LAC presentation	x	x	
Networking	x		
Regional networking	x		
Just go ahead - continue	x	x	
Keep the high quaity		x	x
Keep the familiar spirit and atmosphere			x
More information	x	x	
Information material	x	x	x
Help with questions and personal support	x	x	
Handouts for the participants	x	x	
Collection of ideas	x	x	
I want to support Last Aid	x		
New developments	x	x	
Renewal of the homepage			x
More instructors with different professional backgrounds			x
Giveaways for fundraising			x
A new concept for an LAC for managers			x
Humour as theme in the curriculumn		x	
More education for the instructors and symposia		x	
Continue the research and further development of the curriculum		x	
Morre courses in schools and concept for kids and teens		x	
Inckusion of more about grief and loss		x	
